# Seasonal Affective Disorder: An Overview of Assessment and Treatment Approaches

**DOI:** 10.1155/2015/178564

**Published:** 2015-11-25

**Authors:** Sherri Melrose

**Affiliations:** Faculty of Health Disciplines, Athabasca University, Athabasca, AB, Canada T9S 3A3

## Abstract

Seasonal affective disorder or SAD is a recurrent major depressive disorder with a seasonal pattern usually beginning in fall and continuing into winter months. A subsyndromal type of SAD, or S-SAD, is commonly known as “winter blues.” Less often, SAD causes depression in the spring or early summer. Symptoms center on sad mood and low energy. Those most at risk are female, are younger, live far from the equator, and have family histories of depression, bipolar disorder, or SAD. Screening instruments include the Seasonal Pattern Assessment Questionnaire (SPAQ). Typical treatment includes antidepressant medications, light therapy, Vitamin D, and counselling. This paper provides an overview of SAD.

## 1. Introduction

As sunlight decreases during the short dark days of winter, many individuals struggle with seasonal affective disorder or SAD. As the acronym so aptly illustrates, those afflicted experience feelings of sadness and loss of energy, especially during December, January, and February, around the winter solstice, when the days are shortest. Existing research has focused on the key treatment approaches of antidepressant medication, light therapy, Vitamin D, and counselling. This paper presents an overview of SAD by explaining the disorder and commenting on treatment approaches.

## 2. An Explanation of Seasonal Affective Disorder (SAD)

### 2.1. What Is SAD?

Seasonal affective disorder or SAD is not considered a unique diagnostic entity. Rather, it is a type of recurring major depression with a seasonal pattern. According to the* Diagnostic and Statistical Manual of Mental Disorders* DSM-5 [1], criteria for depression with a seasonal pattern include having depression that begins and ends during a specific season every year (with full remittance during other seasons) for at least two years and having more seasons of depression than seasons without depression over a lifetime. Seasonal pattern disorders occur most frequently in winter although they can also occur in summer.

People with seasonal affective disorder have difficulty regulating the neurotransmitter serotonin, a neurotransmitter believed to be responsible for balancing mood [2]. In one study, people with SAD had 5% more SERT, a protein that assists with serotonin transport, in the winter months than in summer [2]. SERT transports serotonin from the synaptic cleft to the presynaptic neuron, so higher SERT levels lead to lower serotonin activity, thus causing depression [2]. Throughout the summer, sunlight generally keeps SERT levels naturally low [2]. But as sunlight diminishes in the fall, a corresponding decrease in serotonin activity also occurs.

People with SAD may also have difficulty with overproduction of melatonin [3]. Melatonin is a hormone produced by the pineal gland that responds to darkness by causing sleepiness [4]. As winter days become darker, melatonin production increases and, in response, those with SAD feel sleepy and lethargic [5]. Although melatonin likely plays a role in impacting the symptoms of SAD, it cannot by itself account for these phenomena [6].

The combination of decreased serotonin and increased melatonin impacts circadian rhythms. Circadian rhythms or the body's internal 24-hour “clock” are synchronized to respond to the rhythmic light–dark changes that occur daily and throughout each of the seasons. For people with SAD, the circadian signal that indicates a seasonal change in day length has been found to be timed differently, thus making it more difficult for their bodies to adjust [7–9].

Further, with less outdoor exposure to sunlight on the skin in winter, people with SAD may produce less Vitamin D [10, 11]. As Vitamin D is believed to play a role in serotonin activity, Vitamin D deficiency and insufficiency have been associated with clinically significant depressive symptoms [12, 13]. Causal links between serotonin, melatonin, circadian rhythms, Vitamin D, and SAD have not yet been confirmed. However, associations among these key factors are present and are continuing to be researched.

#### 2.1.1. Symptoms

Symptoms of winter seasonal pattern disorders center on sad mood and low energy [14–18]. Information for the lay public identify that people with SAD can feel sad, irritable, and may cry frequently; and they are tired and lethargic, have difficulty concentrating, sleep more than normal, lack energy, decrease their activity levels, withdraw from social situations, crave carbohydrates and sugars, and tend to gain weight due to overeating [5, 19–21].

Conversely, in addition to irritability, symptoms of the less frequently occurring summer seasonal pattern disorder center on poor appetite with associated weight loss, insomnia, agitation, restlessness, anxiety, and even episodes of violent behavior [22, 23]. It is important to note that seasonal pattern disorders vary in severity. Some individuals may experience a milder form of SAD known as subsyndromal S-SAD [24–26], or most commonly as “winter blues.” However, others can be severely incapacitated and unable to function. In some instances, symptoms of SAD can be as severe as those experienced by in-patients with nonseasonal depression [27, 28]. Like all depressive disorders, thoughts of suicide may be present [29, 30]. Health professionals must always implement suicide assessments with people they believe have or might have SAD.

#### 2.1.2. History

Although low mood and low energy levels during the short dark days of winter may have always been an expected part of life for those living far from the equator, they were first identified as a treatable clinical condition during the 1980s [31–34]. When physician Norman Rosenthal moved to the United States from his native South Africa, he noticed that he felt much less productive in the winter but returned to normal as soon as spring arrived [35]. In his work at the National Institutes of Health, NIH, in the US, Rosenthal collaborated with Al Lewy, who was researching melatonin, and with Tom Wehr who was researching how light suppressed melatonin and impacted circadian rhythms. Together, they applied and disseminated their findings about how bright light could effectively treat patients with SAD [31].  The notion of SAD as a depressive condition warranting further study resonated with many who live in northern latitudes and is now a common and well-documented disorder [36].

#### 2.1.3. Prevalence

Seasonal affective disorder occurs four times more often in women than in men and the age of onset is estimated to be between 18 and 30 years [35]. Those living farthest from the equator in northern latitudes are most susceptible [37]. For example, in the United States, 1% of those who live in Florida and 9% who live in Alaska experience SAD [38]. In Canada 15% of the population experience winter blues and 2 to 6% experience SAD [39]. In the United Kingdom, 20% experience winter blues and 2% experience SAD [40, 41].

Pinpointing prevalence is difficult as the disorder may go unreported and consequently underdiagnosed [42]. SAD can cooccur with other depressive, bipolar, attention deficit, alcoholism, and eating disorders, making it difficult to diagnose [43]. As people with SAD may also have subtle decreases in thyroid function, their hypothyroidism can mask symptoms of SAD [44]. Given that SAD is a disorder women often experience and one that is triggered by limited exposure to sunlight, nurses and other health professionals who do shift work may be at particular risk [45].

### 2.2. Seasonal Pattern Assessment Questionnaire (SPAQ)

Clearly a significant number of people are living with the debilitating effects of SAD and are not functioning to their full potential. By screening for SAD and S-SAD, particularly in familiar primary care settings where clients are accustomed to coming for treatment, health professionals can help identify those who are suffering [46].

The Seasonal Pattern Assessment Questionnaire (SPAQ) first developed by Rosenthal and colleagues in 1984 [47] continues to be widely used [31, 48, 49]. The SPAQ is a retrospective, self-administered tool that screens for the existence of SAD and S-SAD. It is freely available in the public domain and can be downloaded from http://www.guilford.com/add/forms/rosenthal2.pdf. No training is required to use the tool.

#### 2.2.1. Scoring the SPAQ

Scoring the SPAQ is not straightforward and clinicians and researchers use the tool in different ways. Questions two and three provide particularly useful information in that they yield a specific number on the Global Seasonality Score or GSS [49–51]. This number or score can immediately communicate whether SAD or S-SAD is likely present and the degree of severity. As such, health professionals can use these two questions to add GSS to their client/patient assessments.

In question two, respondents rate their sleep length, social activity, mood, weight, appetite, and energy level on Likert scales scored from 0 to 4. In question three, respondents rate the degree that seasonal changes are a “problem” (mild, moderate, marked, severe, or disabling).

A GSS of 11 or above and a problem rating of at least moderate are indicative of SAD. A GSS of 9 or 10 and a problem rating of at least mild are indicative of S-SAD.

#### 2.2.2. Reliability, Validity, and Specificity

The SPAQ has been demonstrated to be reliable in that it measures consistently and to be valid in that it measures what it was designed to measure [49, 51–53]. However, it has been criticized for having low specificity, meaning that results may suggest people who do not have SAD will score as though they do [48]. This low specificity may misclassify people with nonseasonal depressions. This misclassification could in turn indicate misleadingly high estimates of prevalence [42, 54].

## 3. Treatment Approaches

Treatment approaches typically include combinations of antidepressant medication, light therapy, Vitamin D, and counselling. The next section provides a brief outline of these.

### 3.1. Antidepressant Medications

SAD, like other depressions, is believed to be associated with a dysfunction in brain serotonin activity. Therefore, second generation antidepressants (SGAs), such as the Selective Serotonin Reuptake Inhibitors (SSRIs), particularly fluoxetine (Prozac), have emerged as promising antidepressant medication treatments [55–57]. In the seminal Canadian study comparing the effectiveness of fluoxetine and light therapy in SAD (Can-Sad), fluoxetine was found to be as effective and as well-tolerated as light therapy and it was more cost-effective [58].

Bupropion (Wellbutrin), another SGA SSRI, has also been widely promoted as an effective medication for treating SAD [59–61]. In the northern US and Canada, one study revealed that beginning bupropion XL 150–300 mg daily early in the season while people were still well did prevent recurrence of seasonal depressive episodes [62].

With any medication treatment, it is important to draw attention to the issue of adverse effects. A Cochrane review of second generation antidepressants (SGAs) and SAD emphasized that insufficient evidence exists to come to any overall conclusions on the use of SGAs for SAD; and the authors noted that up to 27% of participants treated with SGAs for SAD withdrew from the studies early due to adverse effects [63]. Therefore, although antidepressant medication is a viable and often convenient treatment for SAD, especially for those whose symptoms are incapacitating, other options should also be considered.

### 3.2. Light Therapy

Knowing the difference decreased daylight can make in triggering SAD and S-SAD, approaches seeking to replace the diminished sunshine using bright artificial light, particularly in the morning, have consistently showed promise [31, 33, 43, 58, 64–67]. Light therapy is also referred to as Bright Light Therapy (BLT) or phototherapy.

Light boxes can be purchased that emit full spectrum light similar in composition to sunlight. Symptoms of SAD and S-SAD may be relieved by sitting in front of a light box first thing in the morning, from the early fall until spring [68]. In the Scandinavian countries, light rooms, where light is indirect and evenly distributed, are available [66]. Typically light boxes filter out ultraviolet rays and require 20–60 minutes of exposure to 10,000 lux of cool-white fluorescent light daily during fall and winter [67]. This is about 20 times as great as ordinary indoor lighting [38].

Adverse effects of light therapy are usually less severe than those associated with antidepressants. They include eyestrain, increased risk of age-related macular degeneration, headaches, irritability, and difficulty sleeping [5]. Ocular changes and abnormalities are not associated with light therapy [69]. Light therapy should not be used in conjunction with photosensitizing medications such as lithium, melatonin, phenothiazine antipsychotics, and certain antibiotics [69]. In some cases, hypomania and suicidal ideation may occur, especially during the first few days of treatment [70]. Light therapy use should be monitored by a health professional [71].

### 3.3. Vitamin D

A systematic review and meta-analysis concluded that low levels of Vitamin D are associated with depression [10]. Vitamin D concentration is assessed by serum 25-hydroxyvitamin D (25-OH D) levels: with optimal levels at 30 nq/mL; insufficient levels at less than 30 ng/mL; deficient levels at less than 20 ng/mL; and intoxication levels at greater than 150 nq/mL [72]. Low levels of Vitamin D are usually due to insufficient dietary intake or lifestyle issues such as little outdoor exposure to sunshine [11]. During the winter months of November through February, those living about 33 degrees north or 30 degrees south of the equator are not able to synthesize Vitamin D [73].

Many people with SAD and S-SAD have insufficient or deficient levels of Vitamin D, and although no further studies have confirmed the findings, research investigating this association suggests that taking 100,000 IU daily may improve their symptoms [74, 75]. Taking Vitamin D before winter darkness sets in may help prevent symptoms of depression [12]. Adverse reactions or intoxication is rare but could occur from doses of more than 50,000 IU per day [72].

### 3.4. Counselling

Counselling approaches can provide help and support to people with SAD. In one study, six weeks of Cognitive Behavioral Therapy (CBT) provided in group format during two 90-minute sessions per week was as effective as 30 minutes of 10,000 lux of cool-white fluorescent light each morning [76]. An overarching goal of CBT is to break down problems that seem overwhelming and negative patterns by changing the way people think about them [5].

Other forms of counselling for SAD and S-SAD integrate elements of CBT by providing new ways of thinking about sad mood and low energy. When depressive symptoms are not severe, programs that help people improve their diet by limiting starches and sugars; increase their exercise; manage their stress; avoid social withdrawal; and spend more time outdoors are all recommended [5].

On his website, Norman Rosenthal encourages self-counselling by finding ways to reduce the stress that inevitably accompanies the incapacitating symptoms of SAD. He found that Transcendental Meditation (TM), other forms of mindfulness, yoga, walking, and exercise that is personally enjoyable were beneficial [35]. Rosenthal advocates a diet high in proteins, vegetables, unprocessed foods, and complex carbohydrates. He also suggests planning winter trips to sunny locales before winter sets in and people lack the motivation to do so [35].

As the preceding sections explained, SAD is a disorder precipitated by lack of needed exposure to sunlight. Further, most SAD treatment approaches, the exception being antidepressant medications and counselling, are based on increasing people's exposure to bright light. Health professionals can play a critical role in supporting those who live with SAD by seeking to understand the condition more deeply, to integrate assessment tools such as SPAQ into their practice, and to become aware of current evidence-based treatment approaches.

## 4. Conclusion

In summary, this paper provided an overview of SAD and S-SAD or the “winter blues,” explaining what the disorder is in relation to DSM-5 criteria, symptoms, history, and prevalence. People with SAD experience sad moods and low energy to the extent that they are not able to function. Those who live in northern latitudes are most at risk. The self-reported Global Functioning Scores on the Seasonal Pattern Assessment Questionnaire (SPAQ) can immediately communicate people's views about the severity of their illness.

Sunlight plays a critical role in the decreased serotonin activity, increased melatonin production, disrupted circadian rhythms, and low levels of Vitamin D associated with symptoms of SAD. Antidepressant medications offer some relief. However, light therapy, Vitamin D supplements, and counselling approaches are also emerging as effective treatments. This paper calls for health professionals to integrate SAD assessments and treatments into their practice, both with themselves and with those they care about and for.
